# MR Elastography-Based Assessment of Matrix Remodeling at Lesion Sites Associated With Clinical Severity in a Model of Multiple Sclerosis

**DOI:** 10.3389/fneur.2019.01382

**Published:** 2020-01-13

**Authors:** Shuangqing Wang, Jason M. Millward, Laura Hanke-Vela, Bimala Malla, Kjara Pilch, Ana Gil-Infante, Sonia Waiczies, Susanne Mueller, Philipp Boehm-Sturm, Jing Guo, Ingolf Sack, Carmen Infante-Duarte

**Affiliations:** ^1^Charité – Universitätsmedizin Berlin, Corporate Member of Freie Universität Berlin, Humboldt-Universität zu Berlin, and Berlin Institute of Health, Institute for Medical Immunology, Berlin, Germany; ^2^Department of Neurology, Shenzhen University General Hospital, Shenzhen University Clinical Medical Academy, Shenzhen, China; ^3^Berlin Ultrahigh Field Facility, Max Delbrück Center for Molecular Medicine, Berlin, Germany; ^4^Department of Experimental Neurology and Center for Stroke Research, Charité – Universitätsmedizin Berlin, Corporate Member of Freie Universität Berlin, Humboldt-Universität zu Berlin, and Berlin Institute of Health, Berlin, Germany; ^5^NeuroCure Cluster of Excellence and Charité Core Facility 7T Experimental MRIs, Charité – Universitätsmedizin Berlin, Corporate Member of Freie Universität Berlin, Humboldt-Universität zu Berlin, and Berlin Institute of Health, Berlin, Germany; ^6^Department of Radiology, Charité - Universitätsmedizin Berlin, Corporate Member of Freie Universität Berlin, Humboldt-Universität zu Berlin, and Berlin Institute of Health, Berlin, Germany

**Keywords:** magnetic resonance elastography, experimental autoimmune encephalomyelitis, extracellular matrix, fibronectin, gadolinium-based contrast agent, multiple sclerosis

## Abstract

Magnetic resonance imaging (MRI) with gadolinium based contrast agents (GBCA) is routinely used in the clinic to visualize lesions in multiple sclerosis (MS). Although GBCA reveal endothelial permeability, they fail to expose other aspects of lesion formation such as the magnitude of inflammation or tissue changes occurring at sites of blood-brain barrier (BBB) disruption. Moreover, evidence pointing to potential side effects of GBCA has been increasing. Thus, there is an urgent need to develop GBCA-independent imaging tools to monitor pathology in MS. Using MR-elastography (MRE), we previously demonstrated in both MS and the animal model experimental autoimmune encephalomyelitis (EAE) that inflammation was associated with a reduction of brain stiffness. Now, using the relapsing-remitting EAE model, we show that the cerebellum—a region with predominant inflammation in this model—is especially prone to loss of stiffness. We also demonstrate that, contrary to GBCA-MRI, reduction of brain stiffness correlates with clinical disability and is associated with enhanced expression of the extracellular matrix protein fibronectin (FN). Further, we show that FN is largely expressed by activated astrocytes at acute lesions, and reflects the magnitude of tissue remodeling at sites of BBB breakdown. Therefore, MRE could emerge as a safe tool suitable to monitor disease activity in MS.

## Introduction

Multiple sclerosis (MS) is a chronic inflammatory disease of the central nervous system (CNS) that represents the most common cause of non-traumatic disability in young adults. MS is considered to be an autoimmune disease in which self-reactive immune cells gain access to the CNS leading to myelin destruction and neuronal damage and the subsequent formation of multifocal lesions (1). These pathological hallmarks are also characteristic for experimental autoimmune encephalomyelitis (EAE), which is the prototypical model for MS (2).

In MS, clinical relapses correlate with the development of perivenular inflammatory lesions inside the CNS. Lesion monitoring is commonly required to make an accurate diagnosis and to monitor disease progression and response to treatment. Typically, new lesions are visualized using gadolinium-based contrast agents (GBCA) on MRI. During active inflammatory processes, GBCA cross the leaking blood-brain barrier (BBB), enter the CNS parenchyma, and alter the magnetic properties of the tissue, reducing the T1 relaxation time. Generally, the more severe the inflammatory activity, the greater the burden of GBCA-enhancement on post-contrast T1-weighted scans (3). However, GBCA-MRI has limitations due to safety reasons and lack of sensitivity. We previously showed in EAE that certain lesions may remain undetectable by GBCA (4, 5). Furthermore, there are emerging concerns that GBCA may deposit in the tissue after repeated applications (6).

Magnetic resonance elastography (MRE) could represent a promising alternative to the use of GBCA. MRE provides information about the mechanical properties of tissues (7, 8) by analyzing their response to oscillatory shear stress (9). Using MRE, we reported on reduced brain viscoelasticity in patients with clinically isolated syndrome (10) as well as patients with established relapsing-remitting (11) and chronic-progressive (12) MS. Brain tissue softening was also observed in patients with a neuromyelitis optical spectrum disorder (NMOSD) (13). However, the mechanisms underlying brain softening in patients remain elusive. Our aim is therefore to dissect these mechanisms using animal models of neuroinflammation. Application of MRE in the cuprizone mouse model of demyelination demonstrated that brain softening was associated with demyelination (14). The observed reduction of brain viscoelasticity in this model was not related to tissue inflammation. However, in the EAE model of MS, we previously showed that inflammation was associated with reduced brain elasticity on MRE (15, 16).

In the present study, we evaluated the capacity of MRE to reveal acute inflammatory activity and disease severity, comparing sagittal MRE with conventional GBCA-MRI in the relapsing-remitting EAE model. Furthermore, we aimed to clarify the nature of the tissue alterations at lesion sites by examining changes to the extracellular matrix (ECM). For this we concentrated attention on the proteoglycan fibronectin (FN), as it has been shown to accumulate within perivascular lesions, and correlate with the degree of inflammation (17, 18). We thereby endeavor to improve our understanding of pathological changes detected by MRE but not by conventional GBCA-MRI.

## Methods

### Animals

All procedures were approved by the Animal Welfare Department of the State Office of Health and Social Affairs Berlin (LAGeSo), in accordance with national and international guidelines to minimize discomfort to animals (86/609/EEC). Experimental SJL mice (Charles River Laboratories, Sulzfeld, Germany) were housed in the central animal facility of the Charité—Universitätsmedizin Berlin. All animals were kept in a temperature- and humidity-controlled colony room and maintained on a light/dark cycle of 12/12 h with *ad libitum* access to food and water. Over multiple experiments, mice were divided into two groups, EAE (*n* = 25) and non-manipulated healthy control (*n* = 7).

### Experimental Autoimmune Encephalomyelitis (EAE)

To induce EAE (2, 19), SJL mice were immunized subcutaneously with 250 μg proteolipid protein (PLP) peptide 139-151 (purity 95%; Pepceuticals, Leicester, UK) and 800 μg *Mycobacterium tuberculosis* H37Ra (Difco, Franklin Lakes, NJ, USA) emulsified in 100 μl Complete Freund's adjuvant (CFA) and 100 μl phosphate-buffered saline (PBS). Pertussis toxin (250 ng per mouse; List, Biological Laboratories, Campbell, CA, USA) was injected intraperitoneally on the day of immunization (day 0) and again 2 days later (day 2). After immunization, mice were monitored daily for clinical signs and scored as follows: 0, no disease; 0.5, tail paresis; 1, tail paralysis; 1.5, tail paralysis and righting reflex weakness; 2, hind limb paralysis (one limb); 2.5 Hind limb paralisis and paresis of the other hind limb; 3, paraplegia; 4, paraplegia with forelimb weakness or paralysis; 4.5, moribund with tetraparesis or tetraplegia; and 5, dead.

### *In vivo* Scans

*In vivo* MRE and MRI scans were performed as described previously (16) on a 7 T Bruker Pharmascan 70/16 rodent MR scanner (Bruker Biospin, Ettlingen, Germany), running Paravision 5.1 software, with a 20 mm RF quadrature volume head coil (RAPID Biomedical GmbH, Rimpar, Germany). Mice were anesthetized with 1.5–2.0% isoflurane in 30% O_2_ and 70% N_2_O administered via face mask, with continuous respiration monitoring using a pressure-sensitive pad placed on the thorax (Small Animal Instruments Inc., Stony Brook, NY, USA). The animals were placed on a bed with circulating heated water to maintain constant body temperature. In those animals investigated by both MRE and MRI, the MRE measurements were acquired first, followed by GBCA-MRI after 24 h.

### Magnetic Resonance Elastography (MRE)

The MRE images were acquired in one 2 mm midsagittal slice as described previously (16). Mechanical vibration was generated by an air-cooled electromagnetic Lorentz coil in the fringe field of the MRI scanner. Vibrations were initiated by a trigger pulse from the control unit of the MRI scanner, and transferred to the animal through a carbon fiber piston, which was connected to the bite bar transducer. The transducer was gimbaled through a rubber bearing and retaining bracket at the temperature-controlled mouse bed. A plastic disk held up the entire setup in the center of the magnet bore (16).

The timing of the vibration was defined and recorded by a fast low-angle shot (FLASH) sequence especially made for MRE measurement. The direction of the motion sensitizing gradient (MSG), with a strength of 285 mT/m, a frequency of 900 Hz, and 9 periods, was maintained parallel to the principal axis of the magnetic field (maximum amplitude of the mechanical driver was 10 microns). To compensate for the static phase contributions, phase difference images were calculated from two images differing in the sign of the MSG. Frequency amplitude and the number of cycles were controlled by a waveform generator connected via an audio amplifier to the driving coil. Additional scan parameters were as follows: TE = 14.3 ms; TR array = 166.0 ms; slice thickness = 2.0 mm; matrix = 128, FOV = 25 mm; two averages; eight dynamic scans over a vibration period and an acquisition time of 12 min.

### MRE Data Analysis

Complex wave images corresponding to the harmonic drive frequency were extracted by temporal Fourier transformation of the unwrapped phase-difference images. To reduce noise, in addition to the Butterworth band pass filter, a spatiotemporal directional filter was applied to the wave images (20). The spatiotemporal filter filtered waves that were propagating from bottom-to-top in the sagittal slice. A 2D-Helmholtz inversion was performed to the filtered data, yielding the complex shear modulus G^*^ and the magnitude modulus |G^*^| = abs (G^*^). The calculated spatially averaged G^*^-values were represented by the real part of the complex shear modulus G' = Re (G^*^), known as the storage modulus that represents tissue elasticity, and the imaginary part G′ = Im (G^*^), which is the loss modulus representing tissue viscosity. The magnitude, storage and loss moduli were expressed in pascals (Pa). The loss factor calculated as the phase angle phi = arctan (G″/ G′) represents the fluidity of the tissue, which is the degree of viscosity relative to elasticity and is interpreted as being sensitive to the architecture of viscoelastic networks in biological tissues (21). In addition to calculating values for the storage and loss moduli for the entire sagittal slice, the brain was separated into two regions of interest divided at the junction between the cerebrum and the cerebellum.

### Magnetic Resonance Imaging (MRI)

T1 maps were generated using a saturation recovery RAREVTR method, in which the repetition time (TR) was varied to acquire a series of axial T1 weighted images, from which the T1 map was produced. Scan parameters were as follows: TE = 8.3 ms; TR array = 230, 460, 1061, 1485, 2080, 3080 and 7500 ms; flip angle = 90°/180°; RARE factor = 2; slice thickness = 1.0 mm; matrix = 128, FOV = 1.92 cm; NA = 1, 10 slices, scan time = 17 min 44 sec. After acquiring the pre-contrast T1 map, the animals were administered 0.2 mmol/kg gadopentate dimeglumine (Gd-DTPA, Magnevist, Bayer Vital GmbH, Leverkusen, Germany) by intravenous injection. After 5 min, the post-contrast T1 maps were acquired using the same parameters as above. Data acquisition was done with ParaVision 5.1 (Bruker Biospin, Germany). The raw data files were exported as NIFTI image files, and analyzed in ImageJ v. 1.51 (NIH, open source). A region of interest (ROI) defining the brain was manually traced for all 10 slices, and the mean T1 value of each ROI calculated. The mean T1 from all 10 slices was determined for each animal, and the post-contrast mean was subtracted from the pre-contrast mean, to yield the difference—delta T1. The delta T1 was used for the statistical analysis.

In addition, T1-weigthed images were acquired as described previously (22). As a complimentary method, we also calculated the T1 signal intensity change directly from the T1-weighted images. An ROI defining the brain was manually traced for 20 slices and the mean signal intensity (SI) value of each ROI was calculated. The mean SI from all 20 slices was determined for each animal in both pre- and post-contrast, calculated as: signal intensity change (SI%) = [(SI post-contrast—SI pre-contrast)/SI pre-contrast] ^*^ 100.

### Tissue Processing

Mice were sacrificed 1 day after MRE and MRI measurements. Animals were deeply anesthetized with ketamine/xylazine, then transcardially perfused with PBS. Twenty-five brains were extracted and 19 of them were cut sagittally in two symmetrical halves. Half of the brain was postfixed in 4% paraformaldehyde (PFA) overnight at 4°C and then PBS washed, followed by 30% sucrose in PBS, soaking until the tissue sunk to the bottom. Meanwhile, the other half of the brain was reserved for RNA extraction. For histological analysis, brain tissue was embedded in O.C.T., frozen in methylbutane with dry ice, and stored at −80°C. For analysis, frozen tissues were cut into 12 μm coronal or sagittal cryosections and stored at 4°C.

### Histology and Immunofluorescence

For Immunostaining, brain sections were permeabilized and blocked with PBS containing 10% normal goat serum, 10% bovine serum albumin and 0.3% Triton TM X-100 for 1 h at room temperature. Sections were incubated overnight at 4°C with the primary antibodies diluted in PBS. Primary antibodies included: rabbit anti-fibronectin, 1:200 (Millipore ab2033); mouse anti-EIIIA-fibronectin, 1:200 (Abcam ab6328); chicken anti-GFAP, 1:500 (Abcam ab4674). For double labeling immunostaining, primary antibodies were incubated sequentially. For staining with the mouse anti-EIIIA-fibronectin (IST9), we used a mouse on mouse (M.O.M.) kit from Vector Laboratories. After staining with primary antibodies, sections were washed with PBS and incubated with Alexa Fluor (488 or 647, 594)-conjugated secondary antibodies (1:400) at room temperature for 2 h, followed by 4′,6-diamidino-2-phenylindole (DAPI) to visualize cell nuclei. Sections were imaged using a Zeiss Axio Observer fluorescence microscope or a laser-scanned confocal microscope (LSM 710, Carl Zeiss, Jena, Germany).

### Quantitative Reverse-Transcription PCR

The brain tissue for PCR was divided into two portions, the anterior (cerebral) and posterior (cerebellar) region, according to the MRE scanning regions. Total RNA was extracted from the tissue by using the Trizol method. The RNA was reverse transcribed, and quantitative PCR (qPCR) carried out as described previously, using an ABI Prism 7000 SequenceDetection System (Applied Biosystems, Darmstadt, Germany) (16). Primers and probes were from Eurofins MWG Operon (Ebersberg, Germany), and the sequences used were as followed: Fibronectin, forward *5*′*-ATCATTTCATGCCAACCAGTT-3*′, reverse *5*′*-TCGCACTGGTAGAAGTTCCA-3*′, probe *5*′*FAM-CCGACGAAGAGCCCTTACAGTTCCA-3*′TAMRA. Neurocan, forward *5*′*-GGTGTGCGCACTGTGTA-3*′, reverse *5*′*-CATGTTGTGCTGTATGGTGATG-3*′, probe *5*′*FAM-TTCGACGCCTACTGCTTCCGAG-3*′TAMRA. Brevican, forward *5*′*-AGAACCGCTTCAATGTCTACTG-3*′, reverse *5*′*-ACTGTGACAATGGCCTCAAG-3*′, probe *5*′*FAM-ACTCTGCCCATCCCTCTGCTTC-3*′TAMRA. Glypican5, forward *5*′*-GAGACACTTGCCAACAGAAGA-3*′, reverse *5*′*-GGGCAGCCAATTCATTAACAC-3*′, probe *5*′*FAM-CATGGGTCCTTCTATGGTGGCCTG-3*′TAMRA. 18s, served as the endogenous reference, forward *5'-TTCGAACGTCTGCCCTATCAA-3*′, reverse *5*′*-TCCCCGTCACCCATGGT-3*′, probe *5*′*FAM- TGATGTTTATTGACAACACGCTTTACTTTATACCTGAAGA-3*′TAMRA. We used the 2^−ΔΔ*C*_T_^ method to analyze the results.

### Statistical Analysis

Data were analyzed by unpaired two-tailed *t*-test, paired two-tailed *t*-test or repeated-measures analysis of variance (ANOVA), as appropriate. The non-parametric Spearman correlation was used to assess correlation between MRE parameters and EAE score. Pearson correlation was used to assess correlation between imaging parameters, and between MRE parameters and the PCR or immunostaining quantification data. Analysis was done using GraphPad Prism v.5.01. (GraphPad software, La Jolla, CA, USA). ^*^p < 0.05, ^**^p < 0.01, ^***^p < 0.001.

## Results

### During Relapsing-Remitting EAE, Brain Viscoelasticity Is Altered Particularly in the Cerebellum

**Figure 1:**
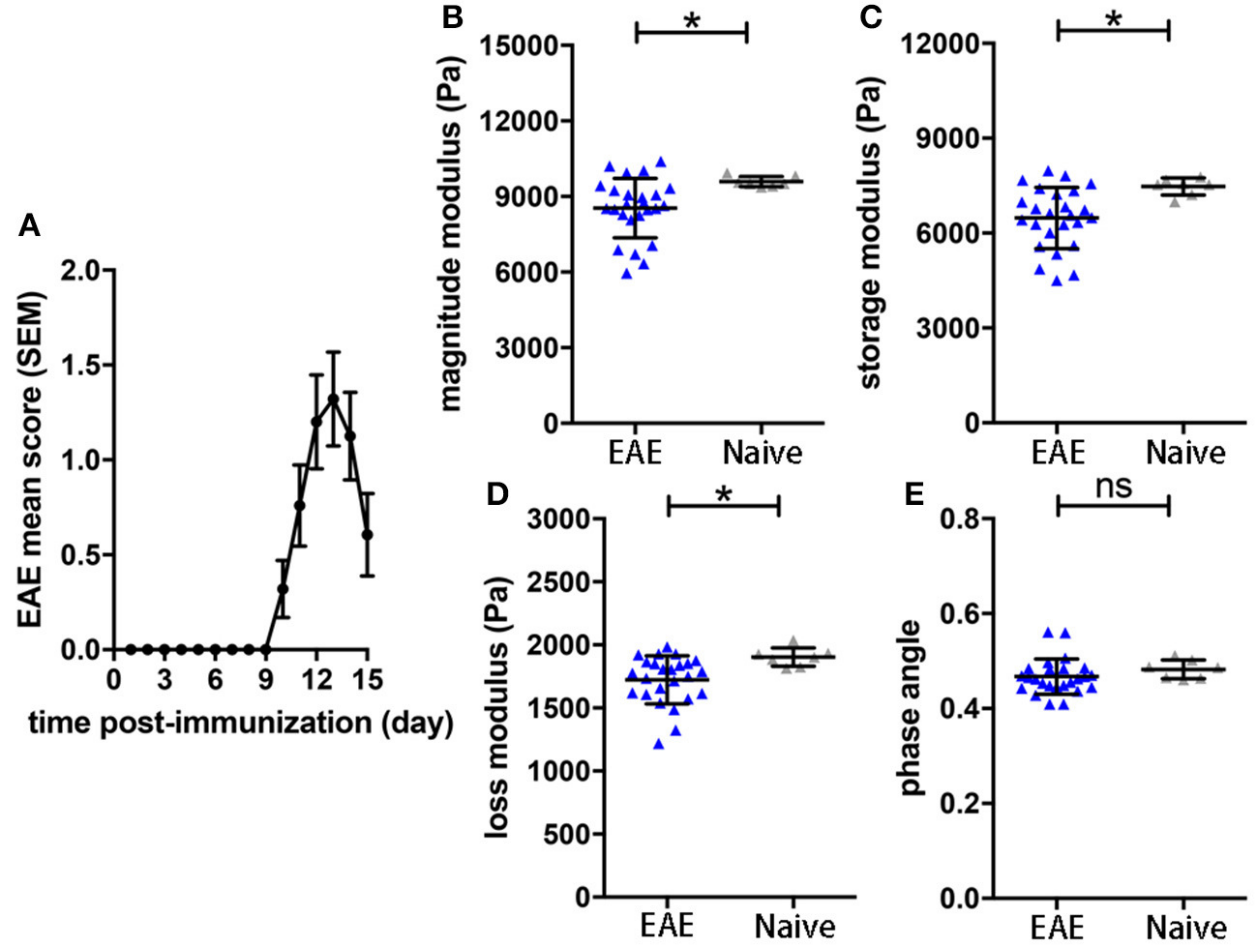
Brain viscoelasticity in EAE and control mice. **(A)** EAE clinical course of SJL mice immunized with PLP. EAE mice reached the maximal score at day 12–14 after immunization, mean with SEM. At days of expected peak disease, EAE mice showed significant reductions of **(B)** magnitude modulus, **(C)** storage and **(D)** loss modulus, compared to healthy controls. **(E)** No alteration of the phase angle was observed in EAE mice. Unpaired two-tailed *t*-test, *p < 0.05, mean ± SD.

To assess the sensitivity of the sagittal MRE protocols in the SJL EAE model (16), we monitored mechanical brain changes in SJL mice immunized with PLP. Mice developed a typical relapsing-remitting disease course, showing first clinical signs by 9–10 days after immunization, reaching peak disease 3–4 days later (day 12-14 p.i.; Figure 1A). MRE measurements were performed at day 14 post-immunization, when clinical signs of disease were well-established and coinciding with the expected disease peak. MRE data were acquired in one 2 mm midsagittal slice and confirmed our previous MRE data in relapsing-remitting EAE acquired in coronal slices (15). We observed that at day 14–15 p.i. the overall viscoelasticity of the tissue (|G^*^| magnitude modulus) as well as the storage modulus G' (elasticity) and the loss modulus G” (viscosity) were significantly diminished in EAE mice compared to controls (Figures 1B–D). No alteration of the phase angle (loss factor) was observed (Figure 1E), indicating that the overall architecture of the brain tissue was not affected by acute inflammation.

**Figure 2:**
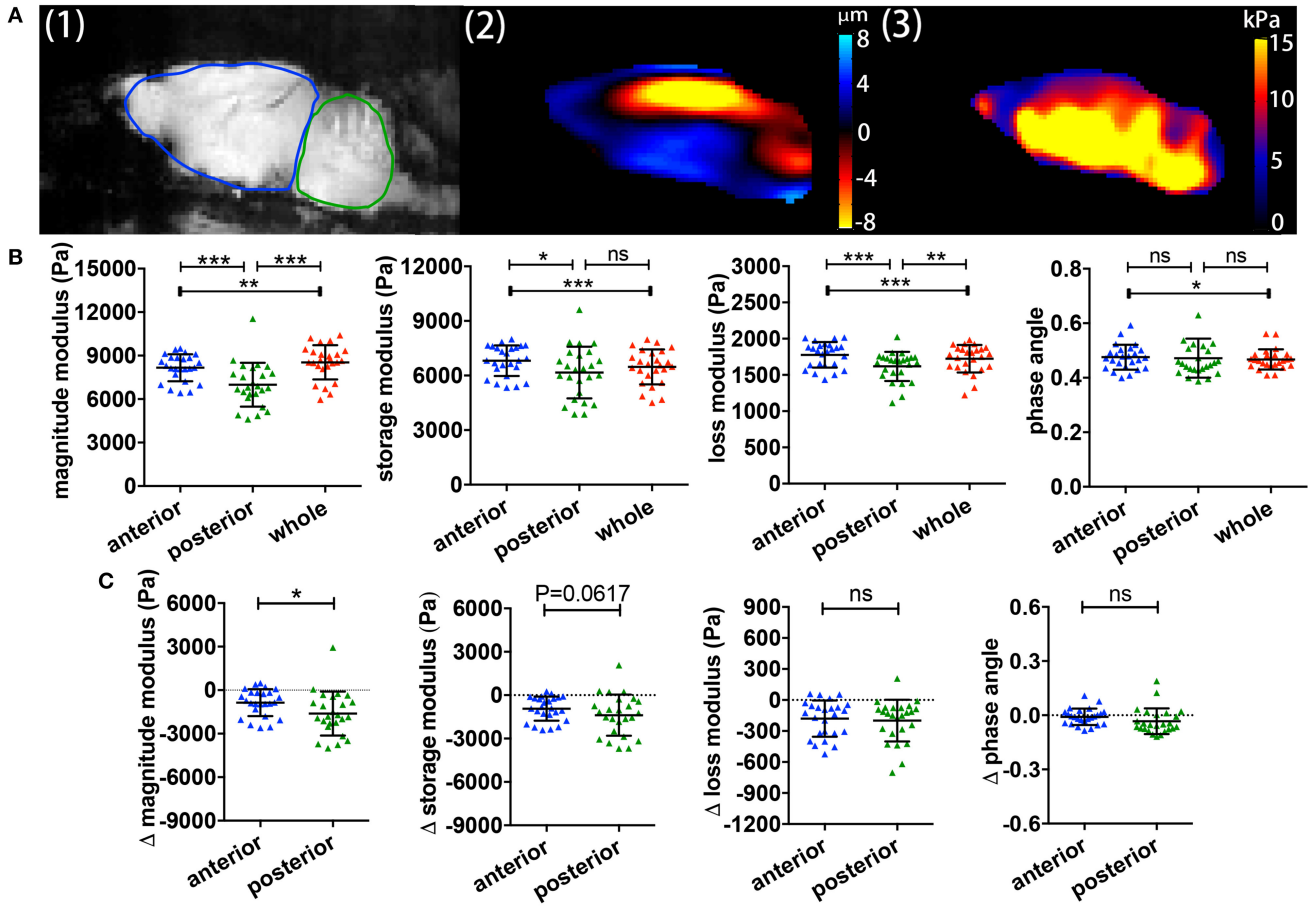
Comparisons of regional brain viscoelasticity. **(A)** Sagittal views of an EAE mouse brain showing a representative magnitude image with definitions of the cerebrum/anterior (red) and cerebellum/posterior (blue) regions (1), a wave (deflection) image (2) and a map of the complex modulus superimposed on the magnitude image (3). **(B)** Comparison of the viscoelastic properties of the posterior/cerebellar region with the anterior region and the whole brain during inflammation. Repeated measures ANOVA, *p < 0.05, **p < 0.01, ***p < 0.001. **(C)** Delta MRE values at the time of the expected disease peak normalized to the values of age- and gender-matched healthy animals. The magnitude modulus—i.e., the tissue rigidity—is particularly diminished in the posterior region (cerebellum) compared to the anterior region (cerebrum). Data from five independent experiments *n* = 25. Paired two-tailed *t*-test, *p < 0.05, mean ± SD.

We previously showed in C57/BL6 mice that different brain regions also show distinct viscoelastic properties (16). However, it remained unclear whether this was also applicable in the SJL EAE model, and whether different brain regions may also differ in the MRE values during relapsing-remitting EAE. A representative example of a midsagittal slice from a SJL EAE mouse is shown in Figure 2A, illustrating the magnitude image A (1), the wave deflection image A (2) and the magnitude of the complex shear modulus |G^*^| A (3). The acquisition of MRE data in midsagittal slices permits the separate analysis of the anterior region (cerebrum) and the posterior brain region (cerebellum) (ROIs in Figure 2A-1). The results show that during EAE, the cerebellum shows a striking reduction of the magnitude, storage and loss moduli, when compared with the cerebrum or the whole brain (Figure 2B). The phase angle remained stable in the investigated regions. Furthermore, to estimate the effect of disease in these two different brain regions, we calculated the viscoelastic changes of the EAE tissue in relation to sex and age matched healthy controls. Figure 2C demonstrates that viscoelastic values at the time of the expected peak of EAE were decreased in both regions when normalized to healthy mice, as indicated by mean difference Δ|G^*^| < 0, indicating that during EAE both regions undergo a “softening” of the tissue. Nevertheless, the magnitude modulus decreased more strikingly in the cerebellum, consistent with the fact that this region is more affected by inflammatory pathology than the cerebrum in the SJL EAE model.

### Sagittal MRE Measurements Show a Correlation Between Mechanical Brain Properties and Clinical Disability in the Relapsing-Remitting EAE Model

**Figure 3:**
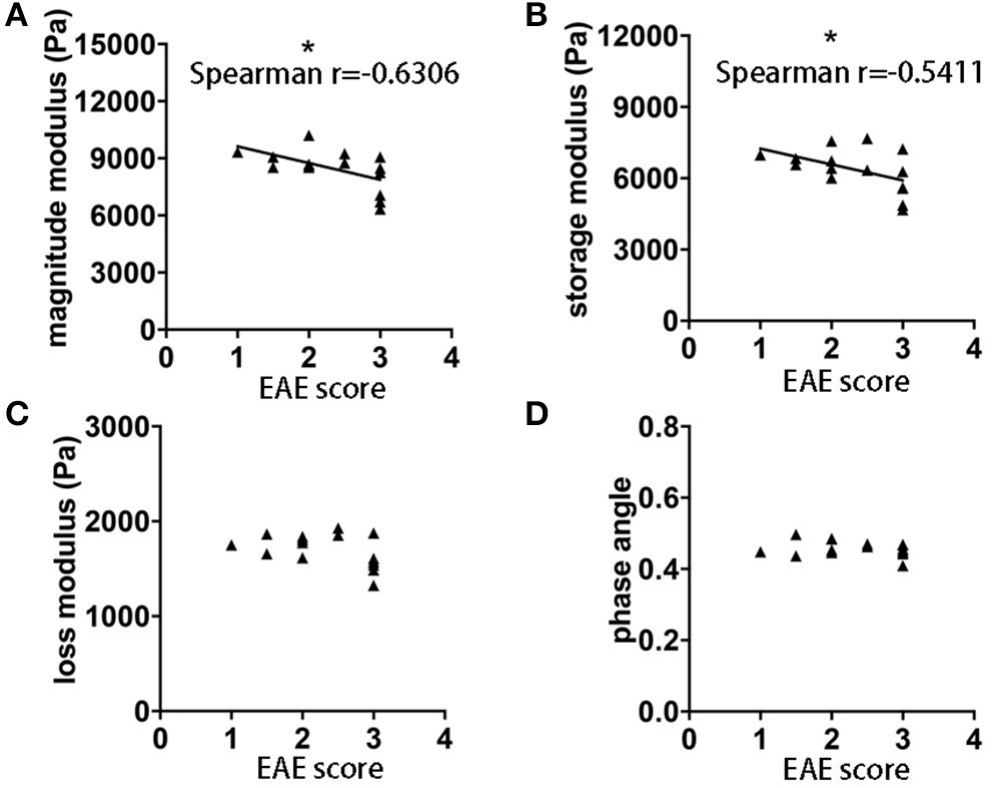
Correlation of EAE clinical score and whole brain viscoelasticity. **(A)** Negative correlation was observed between clinical disability and magnitude modulus (*p* = 0.0117, *r* = −0.6306) as well as **(B)** storage modulus (*p* = 0.0373, *r* = −0.5411). No correlation was observed with **(C)** loss modulus and **(D)** phase angle. Data from five independent experiments, including only symptomatic mice, *n* = 15. Spearman correlation, *p < 0.05.

It is well-established in both MS patients (23) and EAE (24), that MRI measurements do not always correlate with clinical disability. Using MRE acquired in the sagittal plane, we observed statistically significant correlations between the magnitude and storage moduli and the clinical score on the day of MRE acquisition, *p* = 0.0117, *r* = −0.6306 and *p* = 0.0373, *r* = −0.5411, respectively (Figures 3A,B). No significant correlations between loss modulus, phase angle and EAE score were observed (*p* = 0.1211 and *r* = −0.4179, *p* = 0.6189 and *r* = −0.1399, respectively Figures 3C,D). These results indicate that there is an association between brain softening and more severe clinical signs in EAE mice.

### MRE Does Not Correlate With Gadolinium Enhancement in Acute EAE

**Figure 4:**
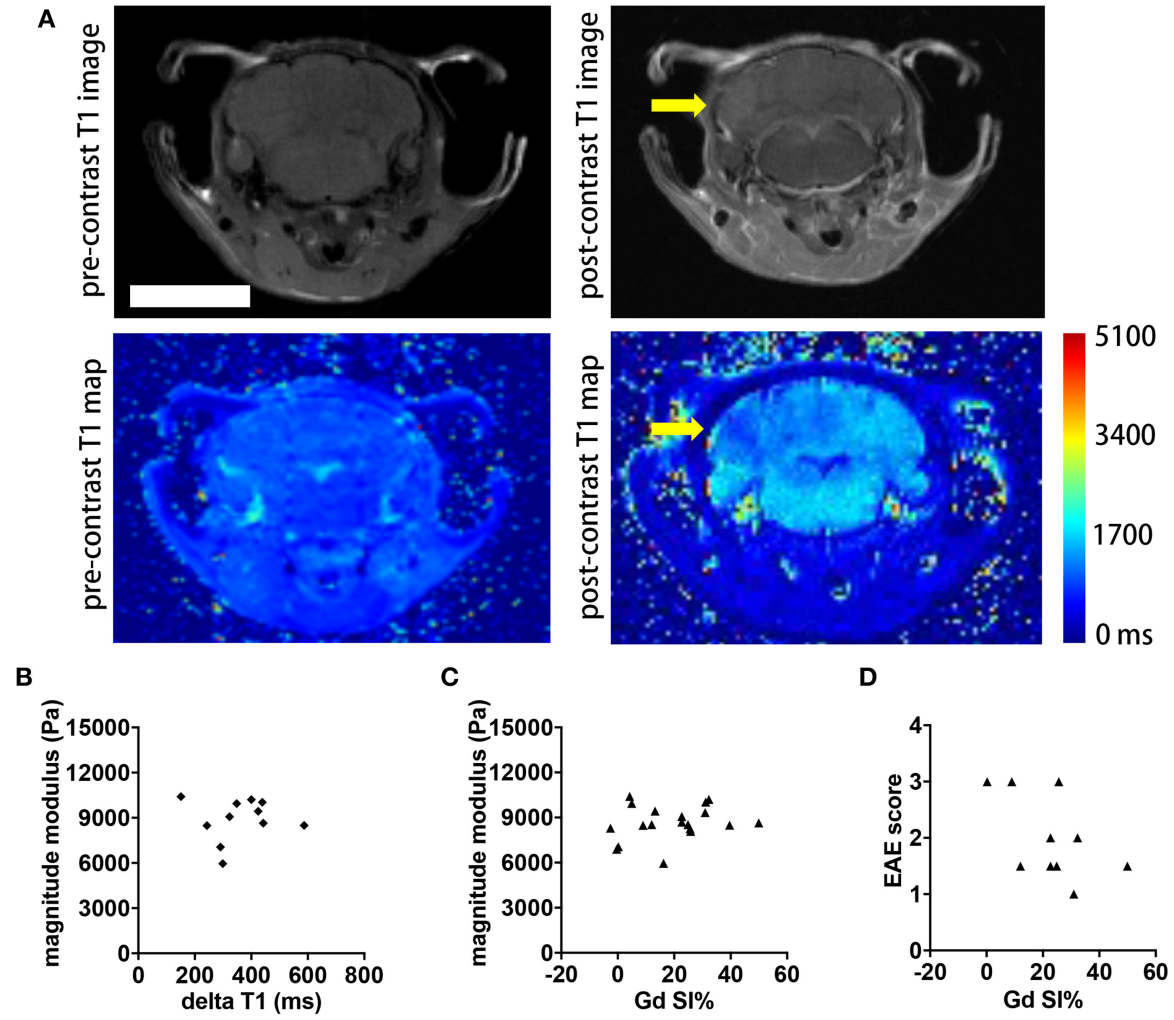
Comparison of whole brain contrast-enhancing lesion burden with whole brain viscoelasticity. **(A)** Representative T1-weighted MR images pre- (left) and post- (right) GBCA administration. A diffuse contrast-enhancing lesion is seen in the cerebellum (arrow). T1 map pre- and post-contrast with T1 relaxation time in ms. Scale bar = 5 mm. **(B)** Delta T1 (mean pre-contrast minus mean post-contrast) did not correlate with the magnitude modulus (Pearson correlation, *p* = 0.8797, *r* = 0.05181). **(C)** T1 signal intensity changes (SI%) obtained from the T1-weighted images following Gd application showed no correlation with the magnitude modulus (Pearson correlation, *p* = 0.3402, *r* = 0.2315). **(D)** There was no significant correlation between SI% and the EAE score (Spearman correlation, *p* = 0.2325, *r* = −0.4195). Data from two independent experiments, *n* = 11 and *n* = 19 in **(B–D)**, respectively.

Contrast agent-based MRI represents the standard MR tool in MS to detect acute inflammatory lesions (25). Therefore, we asked whether the intensity of gadolinium (Gd) enhancement in brain MRI also correlated with clinical disability and MRE values in the SJL EAE model. We acquired axial and coronal T1-weighted images before and after intravenous injection of 0.2 mmol/kg Gd contrast agent, 24 h subsequent to the MRE scans. Brain lesions in active EAE are unevenly distributed in space, and are highly diffuse, lacking clear boarders. In order to better evaluate the extent of these diffuse lesions, we generated T1 maps to yield a quantitative measure of brain tissue contrast changes resulting from GBCA leakage across the blood-brain barrier. The T1 values were averaged from ROIs defining the entire brain, in order to obtain a global metric of Gd enhancement in the whole brain. Representative images illustrating the T1 maps pre- and post-contrast are shown Figure 4A. As expected, the post-contrast T1 values for all animals were significantly reduced, compared to the pre-contrast values (data not shown). Contrary to expectations, there was no statistically significant correlation between the delta T1 and the magnitude modulus (*p* = 0.8797, *r* = 0.05181, Figure 4B) or between T1 signal intensity changes directly obtained from the whole brain T1-weighted images and MRE values (*p* = 0.3402, *r* = 0.2315, Figure 4C). Accordingly, no significant correlation was observed between T1 signal intensity changes and EAE scores of the mice (*p* = 0.2325, *r* = −0.4195, Figure 4D).

### Reduction of Viscoelasticity Correlates With an Increased Expression of FN

**Figure 5:**
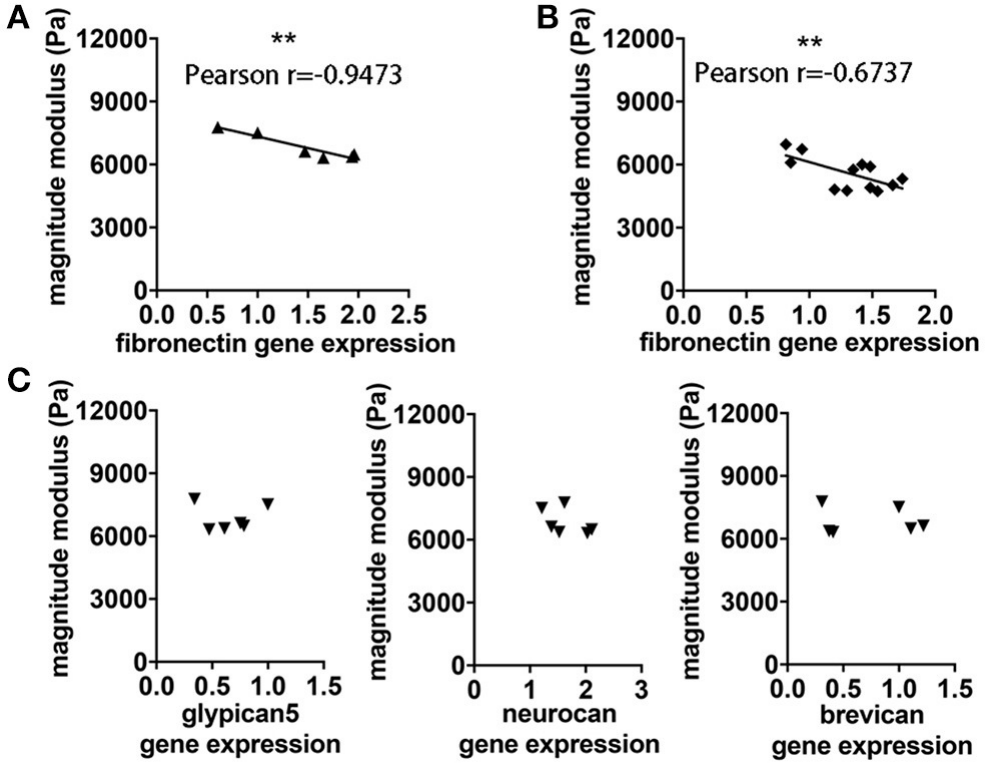
Association between viscoelastic changes and expression of FN. **(A)** The degree of reduction of cerebellar rigidity (magnitude modulus) at time of the expected EAE peak is associated with increased expression of FN assessed by qPCR, *p* = 0.0041, *r* = −0.9473. **(B)** The same association between magnitude modulus and fibronectin expression was found using frozen tissue from our previous study in SJL EAE with coronal MRE. *P* = 0.01163, *r* = −0.6737. **(C)** No correlation was observed between cerebellar magnitude modulus and gene expression of other ECM components of the cerebellum including glypican5, neurocan and brevican, respectively. Pearson correlation, **p < 0.01. Data from two independent experiments, including only symptomatic mice, *n* = 12 and *n* = 6 in **(A–C)**, respectively.

In view of the correlation between acute inflammatory events and loss of brain stiffness as measured by MRE, we considered whether viscoelastic changes may reflect processes of molecular remodeling that occur during the formation of brain lesions. Here we found that reduced viscoelasticity during EAE was significantly correlated with increased gene expression of FN in brain tissue, *p* = 0.0041, *r* = −0.9473 (Figure 5A). This correlation was robust, and was also confirmed in a separate analysis using frozen tissue from our previous study in SJL EAE acquiring MRE data in the coronal orientation (15), *p* = 0.0163, *r* = −0.6737 (Figure 5B).

To assess whether this association may involve changes of other components of the ECM, we investigated the gene expression of other key ECM proteoglycans, associated with the BBB (eg. glypican 5), or with the perineuronal and interstitial matrix (e.g., neurocan and brevican). No significant correlation was observed between brain viscoelasticity and the expression of these key proteoglycans, *p* = 0.9755 and *r* = −0.01632, *p* = 0.3062 and *r* = −0.5056, *p* = 0.8671 and *r* = −0.08886, respectively (Figure 5C).

### FN Deposits Are Predominantly Found in Perivascular Areas, and Reduced Viscoelasticity Correlates With Increased FN Protein Expression

**Figure 6:**
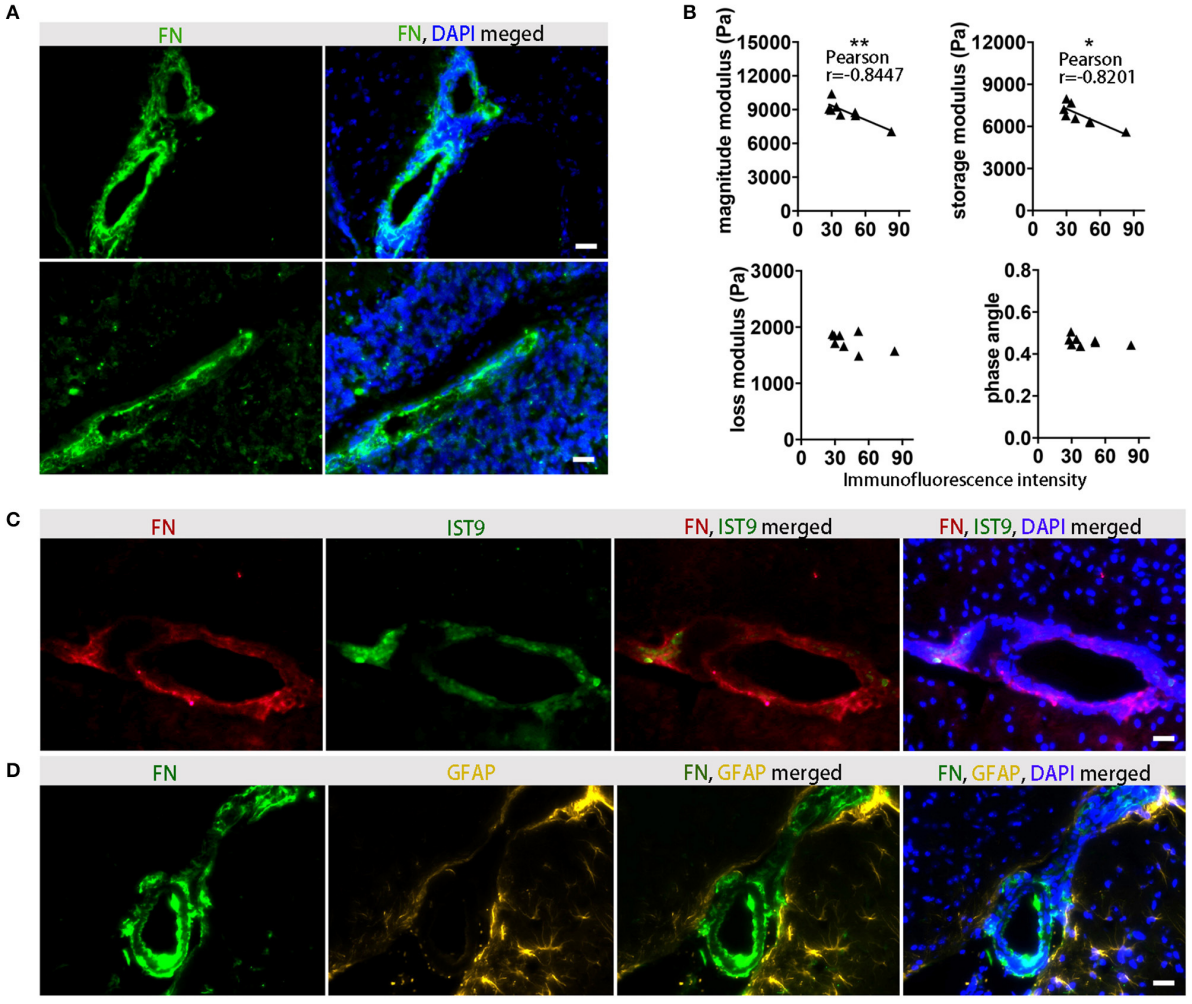
Association between viscoelastic changes and histological detection of FN. **(A)** Immunofluorescence staining indicates a perivascular distribution of FN (green) in inflamed midbrain (top) and cerebellum (bottom). Staining of FN deposits are green and cell nuclei are blue, scale ba *r* = 100 μm. **(B)** Correlation between MRE parameters and FN immunofluorescence in EAE animals at days of expected peak disease. Data from two independent experiments, including only symptomatic mice, *n* = 8. Pearson correlation, **p < 0.01, *p < 0.05. **(C)** Immunostaining with anti-FN for detection of overall fibronectin (red), and anti-EIIIA-FN for detection of cellular FN (IST9, green) indicates that perivascular FN deposits are secreted by cells. Cell nuclei are blue. Scale bar = 100 μm. **(D)** Immunostaining of FN (green) and the astrocytic marker GFAP (yellow) shows perivascular astrocytes adjacent to the FN deposits in the EAE brain. Cell nuclei are blue. Scale bar = 100 μm.

To confirm that FN gene expression is associated with protein deposits, we performed immunohistochemical analysis in the corresponding tissue, and demonstrated that at the time of the expected peak of EAE severity FN was highly expressed in perivascular areas (Figure 6A). In agreement with the gene expression data, reduced viscoelasticity correlated with increased FN immunofluorescence intensity (Figure 6B). In particular, a significant correlation was observed with the magnitude modulus and storage modulus (*p* = 0.0083, *r* = −0.8447 and *p* = 0.0127, *r* = −0.8201, respectively); whereas, no correlation of FN immunostaining with the loss modulus or phase angle could be detected (*p* = 0.1766, *r* = −0.5301 and *p* = 0.2889, *r* = −0.4290, respectively).

FN present in the lesions may arise either from the circulating plasma Fn (pFn), deposited following BBB disruption, or synthesized locally by glial cells (cellular cFn). These two FN variants contain alternatively spliced domains, EIIIA and EIIIB. To clarify the source of the FN observed in EAE, we used an anti EIIIA-FN antibody (IST9), which only recognizes cellular FN, in combination with the broad-spectrum anti-FN antibody. The staining of cellular FN showed a consistently overlapping pattern with the total FN, indicating that the main source of the perivascular FN deposits was indeed cellular, rather than circulating pFn (Figure 6C). The co-staining of FN with the astrocytic marker GFAP further indicated that not only astroglia but also endothelial cells may be major sources of FN production in the lesion sites (Figure 6D).

## Discussion

In this study, we investigated the capacity of MRE to detect acute inflammatory events in the mouse brain during relapsing-remitting EAE. MRE investigations were performed together with T1-weighted MRI using GBCA and were correlated with clinical disability. Furthermore, we assessed the association between MRE values and molecular aspects of extracellular matrix remodeling at the sites of inflammatory lesions.

Studies using MRE to show changes in brain tissue stiffness in the context of pathology should consider the baseline values of normal brain, though it is crucial to take into consideration technical differences among various research groups. For example, Murphy et al. used a higher stimulation frequency (1,500 Hz), yielding higher mouse brain stiffness values (25.0 kPa) (26). Other studies using stimulation frequencies similar to the setup in the current study show that the values we obtained are within the range of other published values [reviewed in Table 1 of Bertalan et al. (27)]. Consistent with our previous studies using coronal MRE (15), we show here that MRE using sagittal slices in SJL EAE revealed a decrease in the overall brain viscoelasticity at the time of the expected peak of disease, compared to the corresponding healthy controls. Furthermore, confirming our data in C57/BL6 mice (16), we demonstrated also in SJL mice that the cerebellum is softer than the cerebrum, and that these regional differences were maintained during EAE. In the SJL model, it is well-documented that the cerebellum is especially susceptible to blood-brain-barrier disruption and lesion formation (28). This together with the significant softening of the cerebellum suggests that areas of high inflammatory activity are especially well-identified by MRE. However, confirming the sensitivity of the sagittal MRE scans for visualizing whole brain inflammation, we observed a significant correlation between whole-brain brain viscoelasticity and EAE clinical score. No correlation was established between cerebellar viscoelasticity and EAE score. Thus, although changes in the cerebellum are relevant and pronounced, they do not seem to reflect all the processes that ultimately determine the clinical EAE signs.

To date, GBCA-MRI represents the standard approach to identify BBB breakdown in MS patients and also in animal models (29–31). However, there is increasing concern from recent studies and case reports pointing out potential toxic side effects of GBCA (32), thus motivating the desire for alternative MRI methods that can both better detect and improve understanding of the nature of the pathology during disease. In the active EAE model, GBCA-enhancing lesions are numerous, but rather small and diffusely distributed, and are thus inherently difficult to quantify using T1-weighted images. Therefore, we used T1 mapping to yield an unbiased, quantitative readout of the burden of lesion activity in the entire brain—a strategy that was recently applied in a mouse brain tumor model (33). Using the delta T1 averaged over the entire brain as a metric of BBB disruption also avoids potential sampling bias that might occur when attempting to quantify lesions in selected brain slices, given the uneven distribution of brain lesions in SJL EAE. Contrary to our expectations, there was no significant correlation between the overall magnitude of GBCA-enhancing lesions and the whole-brain MRE parameters. This underscores the complex nature of multiple pathological processes that occur simultaneously in EAE. It may be that the relationship between viscoelastic changes and GBCA-enhancement cannot be detected with a simple linear correlation. This may reflect that the disease processes that lead to viscoelastic changes have different kinetics from those of acute BBB disruption and active lesions. This remains a topic for further investigations.

Inflammatory lesions are not only defined by enhanced endothelial permeability and disruption of the BBB, but also by astroglial activation and remodeling of the extracellular matrix components (34). In particular FN-mediated signaling seems to promote vascular remodeling during demyelinating disease (35). Therefore, we investigated the relationship between alteration of mechanical properties of the brain and expression of FN, as an indicator for neurovascular junction remodeling during lesion formation. Our data indicate that reduction of brain viscoelasticity is indeed associated with an overall increase of FN expression in the brain tissue (36, 37).

FN is a multidomain glycoprotein binding to cell-surface-receptors, mostly integrins, and to the ECM and appears in two forms: plasma and cellular FN. Cellular FN is secreted as a soluble covalent dimer, and in a complicated process in which the FN molecule undergoes different conformational changes, is assembled into a stable matrix (38). Our data point to a cellular source of the FN deposits detected in the perivascular areas. FN fibers were in close proximity to the reactive astrocytes, confirming previous reports that glia cells are major producers of the protein (39). However, further studies are required to determine the sources of FN. So far, we could not exclude other cellular sources such as brain endothelial cells. Additionally, we observed that FN deposits characterized disturbed BBB with enhanced perivascular space and astrocyte endfoot detachment. Thus, although the FN fibers are extremely elastic (40), and might be expected to contribute to enhanced tissue elasticity, in this context their presence might reflect an assembly of processes involved in disruption of the neurovascular unit at the lesion sites. This may lead to tissue softening, due to enhanced endothelial permeability, alteration of blood flow, enlargement of the perivascular spaces, inflammation or astrocytic endfeed detachment.

Importantly, the correlation of MRE data with FN deposition could also be confirmed in brain tissue from our previous MRE study in SJL animals, in which MRE data was acquired using a coronal slice (15). In contrast, no correlation was found between MRE values and other ECM components such neurocan, brevican, or glypican, which are reported to be relevant for neuroinflammation, but which do not directly reflect the process of acute lesion formation (36, 37). Furthermore, we found that deposits of FN are prominent around inflamed vessels and that the presence of FN also correlated with the overall softening of the brain tissue at acute EAE.

Altogether our study sheds light on the mechanism of brain softening due to inflammation. Previous studies in mouse models and patients have demonstrated *in vivo* that brain tissue becomes softer during the progression of neuroinflammatory processes. Our data demonstrate that some of these observations might be explained by mechanisms involving the enhancement of perivascular spaces and astrocyte endfoot detachment leading to weaker couplings between the neuronal-vascular networks, and reduced tissue stiffness. This might also explain the high sensitivity of MRE to processes of acute inflammation and lesion formation in the brain when compared to GBCA-MRI. The application of MRE in mouse models of brain disease is an emerging field. Future experiments to evaluate how well MRE can predict disease development in the pre-onset phase, or to identify tissue changes and accurately reflect disease severity during clinical relapse and remission, hold promise. Further technical developments that will allow faster acquisition times to obtain MRE data covering the entire mouse brain with reasonable scan times, as well as improvements in spatial resolution will be essential to advance our understanding of the complex processes occurring during neuroinflammation. The results of the present study lay a foundation for such upcoming studies to investigate in more detail the temporal and spatial changes of tissue mechanics in relation with lesion development.

## Data Availability Statement

The raw data supporting the conclusions of this article will be made available by the corresponding author, without undue reservation, to any qualified researcher.

## Ethics Statement

This study was carried out in accordance with the recommendations of national and international guidelines to minimize discomfort to animals (86/609/EEC. The protocol was approved by the Animal Welfare Department of the State Office of Health and Social Affairs Berlin (LAGeSo).

## Author Contributions

CI-D, SWan, JM, LH-V, JG, and IS designed the study. SWan, JM, LH-V, BM, KP, and AG-I carried out the experiments and measurements. SM and PB-S developed the 7 Tesla MRI setup. SWan, JM, JG, BM, and SWai worked on the analysis. SWan, JM, LH-V, and CI-D wrote the manuscript with the assistance of all other co-authors.

### Conflict of Interest

The authors declare that the research was conducted in the absence of any commercial or financial relationships that could be construed as a potential conflict of interest.

## References

[1] Weissert R. The immune pathogenesis of multiple sclerosis. J Neuroimmune Pharmacol. (2013) 8:857–66. 10.1007/s11481-013-9467-3 23660832

[2] Robinson P Harp CT Noronha A Miller SD. The experimental autoimmune encephalomyelitis (EAE) model of MS: utility for understanding disease pathophysiology and treatment. Handb Clin Neurol. (2014) 122:173–89. 10.1016/B978-0-444-52001-2.00008-X 24507518 PMC3981554

[3] Miller DH Grossman RI Reingold SC McFarland HF. The role of magnetic resonance techniques in understanding and managing multiple sclerosis. Brain. (1998) 121:3–24. 10.1093/brain/121.1.3 9549485

[4] Tysiak E Asbach P Aktas O Waiczies H Smyth M Schnorr J. Beyond blood brain barrier breakdown - *in vivo* detection of occult neuroinflammatory foci by magnetic nanoparticles in high field MRI. J Neuroinflammation. (2009) 6:20. 10.1186/1742-2094-6-20 19660125 PMC2731086

[5] Wuerfel E Infante-Duarte C Glumm R Wuerfel JT. Gadofluorine M-enhanced MRI shows involvement of circumventricular organs in neuroinflammation. J Neuroinflammation. (2010) 7:70. 10.1186/1742-2094-7-70 20955604 PMC2978145

[6] Gulani V Calamante F Shellock FG Kanal E Reeder SB International Society for Magnetic Resonance in Medicine. Gadolinium deposition in the brain: summary of evidence and recommendations. Lancet Neurol. (2017) 16:564–70. 10.1016/S1474-4422(17)30158-8 28653648

[7] Bigot M Chauveau F Beuf O Lambert SA. Magnetic resonance elastography of rodent brain. Front Neurol. (2018) 9:1010. 10.3389/fneur.2018.01010 30538670 PMC6277573

[8] Muthupillai R Lomas DJ Rossman PJ Greenleaf JF Manduca A Ehman RL. Magnetic resonance elastography by direct visualization of propagating acoustic strain waves. Science. (1995) 269:1854–7. 10.1126/science.7569924 7569924

[9] Hirsch S Braun J Sack I Magnetic Resonance Elastography: Physical Background And Medical Applications. Weinheim: Wiley-VCH (2017). 10.1002/9783527696017

[10] Fehlner Behrens JR Streitberger KJ Papazoglou S Braun J Bellmann-Strobl J. Higher-resolution MR elastography reveals early mechanical signatures of neuroinflammation in patients with clinically isolated syndrome. J Magn Reson Imaging. (2016) 44:51–8. 10.1002/jmri.25129 26714969

[11] Wuerfel J Paul F Beierbach B Hamhaber U Klatt D Papazoglou S. MR-elastography reveals degradation of tissue integrity in multiple sclerosis. Neuroimage. (2010) 49:2520–5. 10.1016/j.neuroimage.2009.06.018 19539039

[12] Streitberger KJ Sack I Krefting D Pfuller C Braun J Paul F. Brain viscoelasticity alteration in chronic-progressive multiple sclerosis. PLoS ONE. (2012) 7: e29888. 10.1371/journal.pone.0029888 22276134 PMC3262797

[13] Streitberger KJ Fehlner A Pache F Lacheta A Papazoglou S Bellmann-Strobl J. Multifrequency magnetic resonance elastography of the brain reveals tissue degeneration in neuromyelitis optica spectrum disorder. Eur Radiol. (2017) 27:2206–15. 10.1007/s00330-016-4561-6 27572811

[14] Schregel K Wuerfel E Garteiser P Gemeinhardt I Prozorovski T Aktas O. Demyelination reduces brain parenchymal stiffness quantified *in vivo* by magnetic resonance elastography. Proc Natl Acad Sci USA. (2012) 109:6650–5. 10.1073/pnas.1200151109 22492966 PMC3340071

[15] Riek K Millward JM Hamann I Mueller S Pfueller CF Paul F. Magnetic resonance elastography reveals altered brain viscoelasticity in experimental autoimmune encephalomyelitis. Neuroimage Clin. (2012) 1:81–90. 10.1016/j.nicl.2012.09.003 24179740 PMC3757734

[16] Millward JM Guo J Berndt D Braun J Sack I Infante-Duarte C. Tissue structure and inflammatory processes shape viscoelastic properties of the mouse brain. NMR Biomed. (2015) 28:831–9. 10.1002/nbm.3319 25963743

[17] Sobel RA Mitchell ME. Fibronectin in multiple sclerosis lesions. Am J Pathol. (1989) 135:161–8. 2528301 PMC1880224

[18] Stoffels JM de Jonge JC Stancic M Nomden A van Strien ME Ma D. Fibronectin aggregation in multiple sclerosis lesions impairs remyelination. Brain. (2013) 136:116–31. 10.1093/brain/aws313 23365094

[19] Glatigny S Bettelli E. Experimental Autoimmune Encephalomyelitis (EAE) as Animal Models of Multiple Sclerosis (MS). Cold Spring Harb Perspect Med. (2018) 8:a028977. 10.1101/cshperspect.a028977 29311122 PMC6211376

[20] Oliphant TE Manduca A Ehman RL Greenleaf JF. Complex-valued stiffness reconstruction for magnetic resonance elastography by algebraic inversion of the differential equation. Magn Reson Med. (2001) 45:299–310. 10.1002/1522-2594(200102)45:2<299::AID-MRM1039>3.0.CO;2-O 11180438

[21] Sack Jöhrens K Wuerfel J Braun J Structure-sensitive elastography: on the viscoelastic powerlaw behavior of *in vivo* human tissue in health and disease. Soft Matter. (2013) 9:5672–80. 10.1039/c3sm50552a

[22] Millward M Schnorr J Taupitz M Wagner S Wuerfel JT Infante-Duarte C. Iron oxide magnetic nanoparticles highlight early involvement of the choroid plexus in central nervous system inflammation. ASN Neuro. (2013) 5:e00110. 10.1042/AN20120081 23452162 PMC3610189

[23] Barkhof F. The clinico-radiological paradox in multiple sclerosis revisited. Curr Opin Neurol. (2002) 15:239–45. 10.1097/00019052-200206000-00003 12045719

[24] Wuerfel Tysiak E Prozorovski T Smyth M Mueller S Schnorr J. Mouse model mimics multiple sclerosis in the clinico-radiological paradox. Eur J Neurosci. (2007) 26:190–8. 10.1111/j.1460-9568.2007.05644.x 17596194

[25] Kaunzner UW Gauthier SA. MRI in the assessment and monitoring of multiple sclerosis: an update on best practice. Ther Adv Neurol Disord. (2017) 10:247–61. 10.1177/1756285617708911 28607577 PMC5453402

[26] Murphy C Curran GL Glaser KJ Rossman PJ Huston J III Poduslo JF. Magnetic resonance elastography of the brain in a mouse model of Alzheimer's disease: initial results. Magn Reson Imaging. (2012) 30:535–9. 10.1016/j.mri.2011.12.019 22326238 PMC3433281

[27] Bertalan G Guo J Tzschatzsch H Klein C Barnhill E Sack I. Fast tomoelastography of the mouse brain by multifrequency single-shot MR elastography. Magn Reson Med. (2019) 81:2676–87. 10.1002/mrm.27586 30393887

[28] Tonra JR. Cerebellar susceptibility to experimental autoimmune encephalomyelitis in SJL/J mice: potential interaction of immunology with vascular anatomy. Cerebellum. (2002) 1:57–68. 10.1080/147342202753203096 12879974

[29] Pirko Johnson AJ. Neuroimaging of demyelination and remyelination models. Curr Top Microbiol Immunol. (2008) 318:241–66. 10.1007/978-3-540-73677-6_10 18219821

[30] Levy H Assaf Y Frenkel D. Characterization of brain lesions in a mouse model of progressive multiple sclerosis. Exp Neurol. (2010) 226:148–58. 10.1016/j.expneurol.2010.08.017 20736006

[31] Tommasin S Gianni C De Giglio L Pantano P. Neuroimaging techniques to assess inflammation in multiple sclerosis. Neuroscience. (2017) 403:4–16. 10.1016/j.neuroscience.2017.07.055 28764938

[32] Fraum TJ Ludwig DR Bashir MR Fowler KJ. Gadolinium-based contrast agents: a comprehensive risk assessment. J Magn Reson Imaging. (2017) 46:338–53. 10.1002/jmri.25625 28083913

[33] Herrmann K Erokwu BO Johansen ML Basilion JP Gulani V Griswold MA. Dynamic quantitative T1 mapping in orthotopic brain tumor xenografts. Transl Oncol. (2016) 9:147–54. 10.1016/j.tranon.2016.02.004 27084431 PMC4833967

[34] Voskuhl RR Peterson RS Song B Ao Y Morales LB Tiwari-Woodruff S. Reactive astrocytes form scar-like perivascular barriers to leukocytes during adaptive immune inflammation of the CNS. J Neurosci. (2009) 29:11511–22. 10.1523/JNEUROSCI.1514-09.2009 19759299 PMC2768309

[35] Boroujerdi Welser-Alves JV Milner R. Extensive vascular remodeling in the spinal cord of pre-symptomatic experimental autoimmune encephalomyelitis mice; increased vessel expression of fibronectin and the alpha5beta1 integrin. Exp Neurol. (2013) 250:43–51. 10.1016/j.expneurol.2013.09.009 24056042 PMC4235607

[36] Haylock-Jacobs S Keough MB Lau L Yong VW. Chondroitin sulphate proteoglycans: extracellular matrix proteins that regulate immunity of the central nervous system. Autoimmun Rev. (2011) 10:766–72. 10.1016/j.autrev.2011.05.019 21664302

[37] Lorentzen R Melum E Ellinghaus E Smestad C Mero IL Aarseth JH. Association to the Glypican-5 gene in multiple sclerosis. J Neuroimmunol. (2010) 226:194–7. 10.1016/j.jneuroim.2010.07.003 20692050

[38] Schwarzbauer E DeSimone DW. Fibronectins, their fibrillogenesis, and *in vivo* functions. Cold Spring Harb Perspect Biol. (2011) 3:a005041. 10.1101/cshperspect.a005041 21576254 PMC3119908

[39] Stoffels JM Hoekstra D Franklin RJ Baron W Zhao C. The EIIIA domain from astrocyte-derived fibronectin mediates proliferation of oligodendrocyte progenitor cells following CNS demyelination. Glia. (2015) 63:242–56. 10.1002/glia.22748 25156142 PMC4737254

[40] Ohashi T Kiehart DP Erickson HP. Dynamics and elasticity of the fibronectin matrix in living cell culture visualized by fibronectin-green fluorescent protein. Proc Natl Acad Sci USA. (1999) 96:2153–8. 10.1073/pnas.96.5.2153 10051610 PMC26752

